# DNA replication and the GINS complex: localization on extended chromatin fibers

**DOI:** 10.1186/1756-8935-2-6

**Published:** 2009-05-14

**Authors:** Stephanie M Cohen, Paul D Chastain, Marila Cordeiro-Stone, David G Kaufman

**Affiliations:** 1 Department of Pathology and Laboratory Medicine, University of North Carolina, Chapel Hill, North Carolina, USA

## Abstract

**Background:**

The GINS complex is thought to be essential for the processes of initiation and elongation of DNA replication. This complex contains four subunits, one of which (Psf1) is proposed to bind to both chromatin and DNA replication-associated proteins. To date there have been no microscopic analyses to evaluate the chromatin distribution of this complex. Here, we show the organization of GINS complexes on extended chromatin fibers in relation to sites of DNA replication and replication-associated proteins.

**Results:**

Using immunofluorescence microscopy we were able to visualize ORC1, ORC2, PCNA, and GINS complex proteins Psf1 and Psf2 bound to extended chromatin fibers. We were also able to detect these proteins concurrently with the visualization of tracks of recently replicated DNA where EdU, a thymidine analog, was incorporated. This allowed us to assess the chromatin association of proteins of interest in relation to the process of DNA replication. ORC and GINS proteins were found on chromatin fibers before replication could be detected. These proteins were also associated with newly replicated DNA in bead-like structures. Additionally, GINS proteins co-localized with PCNA at sites of active replication.

**Conclusion:**

In agreement with its proposed role in the initiation of DNA replication, GINS proteins associated with chromatin near sites of ORC binding that were devoid of EdU (absence of DNA replication). The association of GINS proteins with PCNA was consistent with a role in the process of elongation. Additionally, the large size of our chromatin fibers (up to approximately 7 Mb) allowed for a more expansive analysis of the distance between active replicons than previously reported.

## Background

In eukaryotes, the process of DNA replication occurs in the S phase of the cell cycle in a highly coordinated manner: it begins with initiation at a few origins of replication, leading to a cascade of origin activation and DNA replication until the entire genome is faithfully duplicated. While the process of replication occurs exclusively in S phase, the framework for this process is laid out much earlier in the cell cycle. Briefly, at the end of mitosis and into the G_1 _phase of the cell cycle, origin recognition complex proteins (ORCs) are assembled together on chromatin. Minichromosome maintenance (MCM) complex proteins 2 to 7 are loaded in a Cdt1- Cdc18/Cdc6-dependent manner to form the pre-replicative complex (pre-RC). At the G_1_/S border, Cdc7 and Cdk2 promote the recruitment of GINS and Cdc45 to pre-RCs, which in turn activate the MCM complex (reviewed in DePamphilis *et al*. [1]). In yeast, Sld3 is also required for this process, but to date no human ortholog has been found [2]. The activation of the MCM helicase, in conjunction with perhaps 20 other cell cycle-related proteins, leads to the start of DNA replication [3]. The GINS complex and Cdc45 stay associated with the MCM complex as DNA is unwound during the elongation phase of DNA replication [4,5]. In humans, MCM proteins as well as ORC1 come off of the chromatin as part of a process that prevents rereplication of DNA sequences, which could lead to amplification of genome regions and genomic instability (reviewed in DePamphilis [3]).

Immunofluorescence (IF) of incorporated nucleotide analogs has been used to map the reproducible punctate patterns of DNA replication in interphase nuclei as cells traverse the S phase [6-11]. The distribution of some DNA replication-associated proteins, such as ORCs and MCMs, has also been studied in interphase nuclei using IF [12-16]. Together, these studies provided insight into the spatial complexity of the DNA replication process. For example, sites of replication are not uniformly distributed across the nucleus; they are found in discrete structures called foci that are composed of an average of approximately 10 replicons [17,18]. In S phase, ORCs and MCMs are detected in close proximity to, but not overlapping, replication foci [12-16]. There is evidence that ORCs and MCMs dissociate from the bulk of the chromatin as cells progress through to the end of S phase, a time when they can still be found associated with late replicating heterochromatin [13,15,16].

The recently discovered GINS complex is a 90-kDa heterotetramer; it is composed of four evolutionarily conserved subunits, namely Sld5 (synthetic lethal with *dpb11 *mutant-5), Psf1 (partner of *sld5*-1), Psf2 and Psf3, and resembles a trapezoid. Each subunit is roughly one quarter of the trapezoid. Sld5 and Psf1 heterodimerize to form the top of the complex and Psf2 and Psf3 the bottom [19]. The center of this complex has high negative electrostatic potential, making it unlikely that GINS has a DNA clamp-like function as was previously proposed [19,20]. In addition to its function in activating the MCM complex, *in vitro *experiments have found that the GINS complex physically interacts with and markedly stimulates the polymerase function but not the priming function of DNA polymerase/primase α [21]. Although structural and biochemical characterization studies of the GINS complex have been reported, to date there have been no microscopic analyses to determine the nuclear distribution of this complex. Here, we show the organization of GINS complexes on extended chromatin fibers in relation to sites of DNA replication and replication-associated proteins. We chose to use extended chromatin fibers instead of interphase nuclei because it complemented our studies of DNA replication dynamics on straightened and aligned DNA fibers [22-24].

Extended chromatin fibers from animal cells have been used to show the distribution of covalently modified histones in some silent chromatin sequences [25] and the organization of associated histones and DNA replication timing in centromeric regions [26-28]. In addition, chromatin fibers from plant cells have been utilized to study DNA replication [29] and for high-resolution fluorescence *in situ *hybridization (FISH) studies [30]. In the present study, extended chromatin fibers were prepared from logarithmically growing normal human fibroblasts, following a 20-min incubation with 5-ethynyl-2'-deoxyuridine (EdU). Indirect IF with primary antibodies to GINS proteins (Psf1, Psf2), ORC1, ORC2, Cdc6 and proliferating cell nuclear antigen (PCNA) was used to localize the sites of attachment to chromatin of these proteins. With the exception of Cdc6, they were all visualized on the extended chromatin fibers. In agreement with its proposed role in the initiation of DNA replication, we found the GINS complex associated with chromatin near sites of ORC binding that were devoid of EdU (absence of DNA replication). GINS proteins were also detected at sites of active replication along with PCNA, as would be expected for proteins involved in the process of DNA elongation. These findings are consistent with published biochemical studies, thus indicating that the methodology used here can be useful in furthering our understanding of protein interactions in the process of DNA replication.

## Results

### Estimating chromatin fiber length and compaction

**Figure 1:**
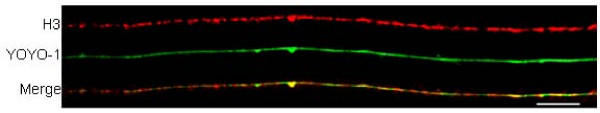
**Visualization of histone H3 on extended chromatin fibers**. Extended chromatin fibers were immunostained for the nucleosome protein histone H3. DNA was counterstained with the DNA dye YOYO-1 (green). H3 (red) was found distributed along the entire chromatin fiber. Bars ≅ 25 μm (≅ 400 kb; bottom right of panel).

Our method for generating extended chromatin fibers resulted in a variety of nuclear structures with chromatin bundles of different thicknesses due to differential lysis. For analyses, we chose the thinnest (smallest diameter) fibers that still had associated proteins. We performed indirect IF with an antibody to histone H3 to determine whether chromatin-associated proteins remained attached to our extended chromatin fibers (Figure 1). DNA was counterstained with YOYO-1. We found that histone H3 was distributed along the entire length of the chromatin fiber indicating that nucleosomes did remain attached.

In order to estimate the average length of DNA (kb) per micron of chromatin fiber, we first determined the conversion factor from length in microns (in digital photomicrographs) to kilobases of straightened and aligned DNA fibers on a microscope slide [22]. FISH was used to probe a region of 282,984 bp in the DNA fiber spreads, and the hybridized region was measured [23]. We used DNA fibers for this analysis because we have been unable as of yet to achieve sufficiently specific FISH signal when using chromatin fibers. We found an average of 1,931 bp/μm with a standard error of the mean (SEM) of 61 bp (based on a total of 13 measurements). Next, we determined the differences in the degree of compaction between DNA spreads and extended chromatin fibers. Normal human fibroblasts were incubated with 30 μM EdU or IdU for 20 min to identify sites of DNA replication. Samples from duplicate cell culture plates were used to either spread and align DNA molecules on glass slides [22] or to prepare extended chromatin fibers. We then measured the track length of sites of DNA replication in the aligned DNA molecules and compared it with the average length of replication tracks in the extended chromatin fibers. We found that after 20 min of labeling the average replication track length in the DNA spreads was 31.9 μm (SEM = 0.7, *n *= 367 tracks) and in chromatin fibers the average length was 3.84 ± 0.15 μm (*n *= 434 tracks from a total of 42 chromatin fibers), giving us a compaction factor of 8.3 and an average of 16 kb of DNA per micron of chromatin fiber. Results from DNA spreads also indicated that an average of 61.5 kb of DNA was replicated in 20 min, or a replication rate of 3.1 kb/min. This is within the range of replication fork movement rates reported for diploid human fibroblasts (0.6 to 3.6 kb/min) [31].

### Distribution of ORC1 and ORC2 on chromatin fibers

**Figure 2:**
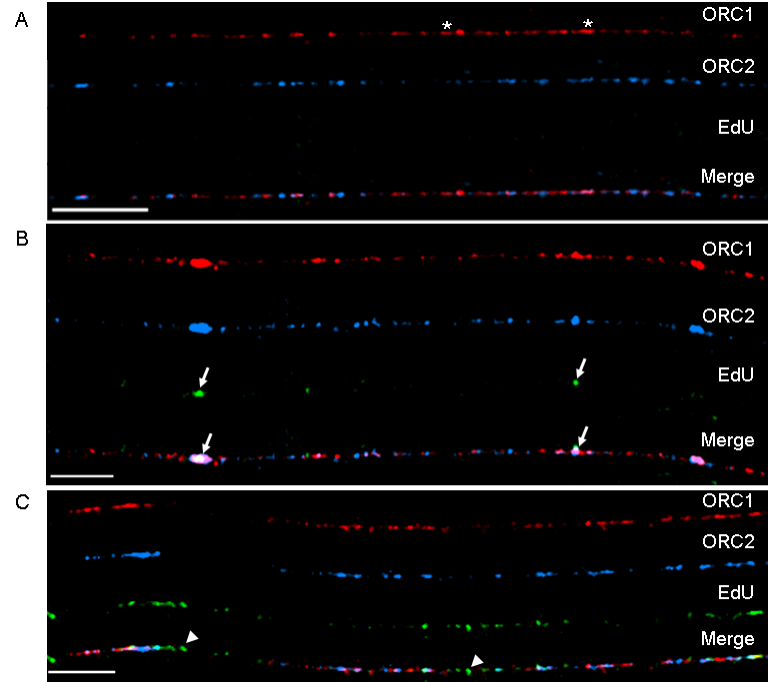
**Distribution of ORC1, ORC2, and EdU tracks**. (A) This chromatin fiber has an abundance of ORC1 (red signal) and ORC2 (blue signal) proteins, but no EdU tracks. Much of the ORC1 signal appears to be found in streaks (asterisks) rather than in a punctate pattern, indicating multiple copies of this protein are present at these sites. The (B) fiber has regions that appear 'bead-like' (arrows), where DNA replication activity is marked by green EdU labeling. In panel (C), several areas undergoing replication have no overlapping ORC1 or ORC2 proteins (two are marked with arrow heads). These most likely represent sites where ORCs have dissociated from the chromatin. Bars ≅ 25 μm (≅ 400 kb; bottom left of each panel).

We used IF to visualize the location of ORC1, ORC2 and EdU tracks on chromatin fibers (Figure 2). The ORC antibodies utilized in these experiments have been previously used to study the binding of cell cycle-related proteins at human origins of replication using chromatin immunoprecipitation (ChIP) technology [32,33]. We found many chromatin fibers with no visible replication activity during the 20 min of EdU labeling (Figure 2A). As ORCs are found on chromatin in G_1 _(before DNA replication begins) this result was not unexpected. ORC1 was found at an average density of one ORC1 signal per 138 kb, and ORC2 was present with an average density of one ORC2 per 188 kb (20 fibers, approximately 80.6 Mb total, 585 ORC1 sites, 429 ORC2 sites). The ratio of ORC2 to ORC1 was 73%. Many of these ORC sites, however, are no doubt comprised of multiple ORC proteins. Often these signals were found in streaks rather than a punctate distribution (Figure 2A, asterisks).

As replication occurred, ORC1 and ORC2 could be found overlapping sites of EdU, as well as between EdU tracks. Sites of overlap were often found in 'bead-like' structures (arrows in Figure 2B), which appear to be covered by ORCs, although these proteins are supposed to be bound at origins of replication (most likely located at the center of the EdU tracks). Our data also suggest that ORCs dissociate from the chromatin associated with the EdU tracks. This interpretation was based on visual evidence (Figure 2C, arrow heads) that was reinforced by the analysis of 18 chromatin fibers to determine the number of EdU tracks that overlapped with ORC1 and ORC2. We found that ORCs overlapped EdU tracks 62% of the time (*n *= 292) leaving 38% of EdU tracks without overlapping ORC1 or ORC2. If we look more closely at the EdU tracks containing ORCs, we find that 38% included both ORC1 and ORC2 signals, 19% showed only the signal from ORC1, and 5% only the ORC2 signal. These differences in frequency of ORC1 and ORC2 signal overlap with EdU tracks could reflect differences in the affinities of their respective antibodies, or differences in the availability of the epitopes recognized by these antibodies, and not the absence of one or the other component of the chromatin-bound complex of ORC proteins. Alternatively, one of the proteins (ORC2 more often than ORC1) might dissociate from chromatin before the other at some locations, or some of the ORC1 binding sites might reflect function(s) for this protein other than in DNA replication initiation.

### Distribution of GINS on chromatin fibers

**Figure 3:**
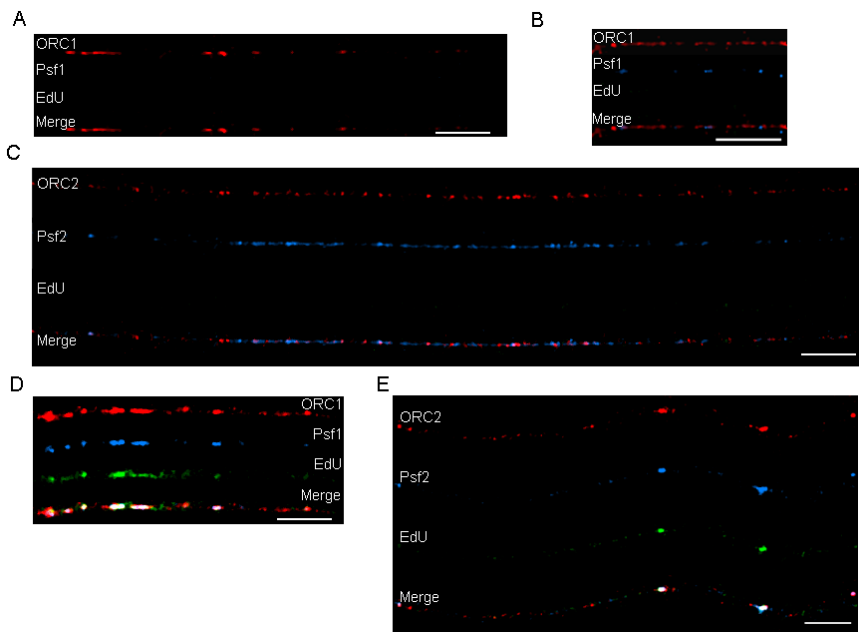
**Distribution of ORCs, GINS and EdU on extended chromatin fibers**. (A) Only ORC1 (red signal) is found on the chromatin fiber; there is no GINS or EdU signal on this fiber. (B and C) Both ORCs (red signal) and GINS (blue signal) can be found along the fiber but no EdU. (D and E) ORCs, GINS complexes and EdU (green signal) are all present. The localization of ORCs, GINS and DNA replication tracks on these fibers suggests that ORCs are loaded on the chromatin first, then GINS, followed by DNA replication. Bars ≅ 25 μm (≅ 400 kb; bottom right of each panel).

We found chromatin fibers that had ORCs but with neither GINS nor EdU tracks present. Before GINS complexes bind and DNA replication is initiated, chromatin fibers are expected to contain ORCs in pre-RCs (Figure 3A). We also found chromatin fibers where ORC and GINS proteins were both bound to chromatin before there was any visual evidence of DNA replication. This could be seen with chromatin fibers stained for either ORC1 or ORC2 (Figures 3B and 3C). The appearance of GINS proteins on chromatin before DNA replication begins is consistent with its proposed role in the initiation of DNA replication [4]. At this stage, as seen in Figure 3C, 36% of GINS overlapped with ORCs overall (*n *= 8 fibers, 137 GINS sites). Once replication began, however, GINS could be found overlapping ORCs at sites of DNA replication 90% of the time at bead-like structures (Figures 3D and 3E).

**Figure 4:**
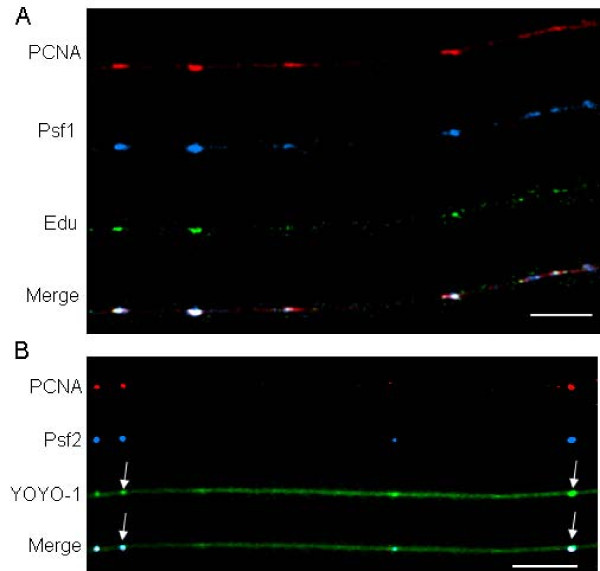
**Distribution of PCNA and GINS on extended chromatin fibers**. (A) There is a high degree of overlap between PCNA (red signal), GINS (blue signal) and EdU tracks (green signal). (B) PCNA (red signal) and GINS (blue signal) coincide with bead-like structures that can also be seen on the DNA (green DNA stain). Two of the bead-like structures are marked with arrows. Bars ≅ 25 μm (≅ 400 kb; bottom right of each panel).

We also examined the distribution of the DNA polymerase clamp PCNA. In IF images of interphase nuclei PCNA is typically found at replication foci along with DNA polymerases, nucleases such as Fen 1, and DNA ligase. Our data showed that PCNA was indeed found at sites of active replication on chromatin fibers (Figure 4A). As predicted from biochemical studies that indicated a role for the GINS complex in the process of elongation [5], we found GINS proteins associated with sites of PCNA and EdU at bead-like structures (Figure 4).

### Distribution of ORCs and GINS complex proteins on chromatin fibers after longer EdU incubation times

**Figure 5:**
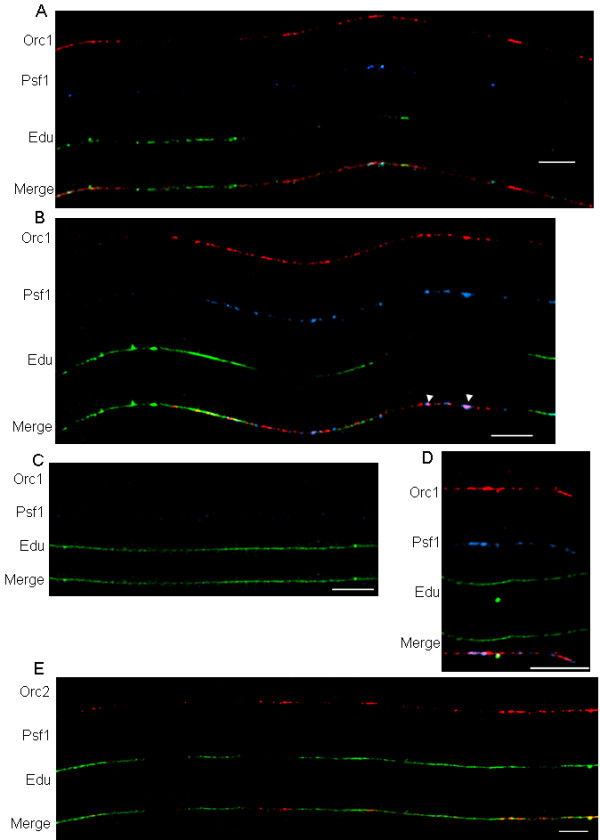
**Distribution of ORCs and GINS on extended chromatin fibers with longer EdU labeling times**. (A to D) ORC1 (red signal), Psf1 (blue signal), EdU tracks (green signal); (E) ORC2 (red signal), EdU tracks (green). (A) Chromatin fiber from cells labeled with EdU for 30 min. (B to E) Chromatin fibers from cells that were labeled for 40 min with EdU. As replication proceeds, the signals from ORCs and Psf1 are no longer visible on chromatin fibers. We also demonstrate here that the depletion of ORC and GINS complex proteins from some fibers is specific and not the result of uneven antibody staining or differences in microenvironments on the slide. This specific depletion can be seen in Panel B, where there are unreplicated regions that still have associated ORC1 and Psf1 proteins (arrow heads) on the same fiber with regions that have completed replication and are devoid of these proteins. Also, in Panel D we show two adjacent fibers, one fiber with immunofluorescent signal from ORC1 and Psf1 but with no EdU staining, the other fiber with only EdU staining and no ORC1 and Psf1 antibody staining. Bars ≅ 25 μm (≅ 400 kb).

The presence or absence of ORCs and GINs bound to newly replicated chromatin was re-examined after labeling cells with EdU for 30 or 40 min. As expected, with 30-min labeling we observed longer EdU tracks (Figure 5A). After 40-min labeling we found even longer tracks (Figures 5B to 5E), some of which covered an entire fiber (Figure 5C). We also looked at the distribution of ORC1 and Psf1 (Figures 5A to 5D) and ORC2 (Figure 5E) on these fibers. Our results were consistent with the interpretation that the overlap of IF signals from these three proteins with EdU decreases as replication proceeds across a chromatin fiber, and regions that have completed replication lack these proteins all together (Figures 5C and 5D). In addition, Figure 5D shows two fibers present in the same field of view, one containing ORC1 and Psf1 with very low amounts of EdU, the other fiber visibly devoid of these proteins, but fully replicated. These results indicate that the absence of IF signal for the ORC and GINS proteins on the replicated fibers is not likely to be due to localized problems with antibody binding.

### Distance between active origins of DNA replication

**Figure 6:**
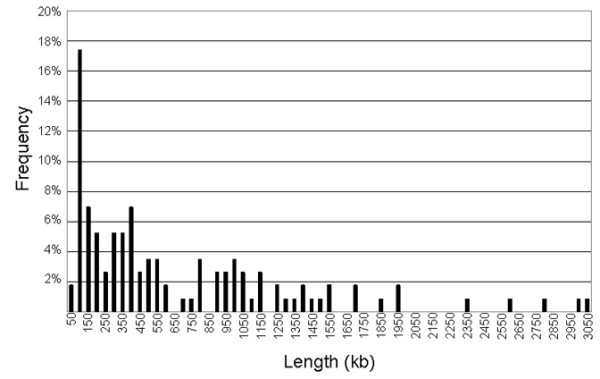
**Distribution of distances between centers of active replication in chromatin fibers**. We measured between the centers of adjacent EdU tracks that overlapped with GINS proteins (*n *= 116) to determine the average distance between sites of active replication. A total of 24 fibers were used for this analysis ranging in size from approximately 1.1 to 7.4 Mb. The frequency distribution is shown in this chart. Numbers on the X-axis represent the upper limit of each bin. We found that the average distance was 667 kb with standard error of the mean of 66. Minimum size = 35 kb, maximum size = 3,505 kb, median 390 kb.

Using chromatin fibers labeled for 20 min with EdU, we measured the distance between the centers of adjacent EdU tracks that also contained GINS proteins (*n *= 116). A total of 24 fibers were used for this analysis, ranging in size from approximately 1.1 to 7.4 Mb (average approximately 4 Mb). The frequency distribution of the measured distances is shown in Figure 6. The highest frequency was for distances between 51 and 100 kb, which could have been measured between neighboring replicons within the same cluster of replication units. Larger distances are more likely to reflect simultaneous replication at different clusters. The average distance of 676 ± 66 kb is longer than was previously reported in studies using autoradiography or IF labeling of adjacent tracks in DNA spreads. The longer distances between active replication sites reported here would not have been measured in combed DNA molecules [34], which are usually 500 kb or smaller. On the other hand, the methodology used in this study would not detect small distances (< 20 kb) between neighboring replicons, as these adjacent EdU-labeled regions would be seen on the chromatin fibers as one large EdU track rather than two. The measurements reported here, therefore, likely represent a combination of distances between origins located within the same replicon cluster (origins located in close proximity that fire simultaneously) and between different replicon clusters that are activated at the same time.

## Discussion

The process of DNA replication involves a multitude of proteins that need to be loaded onto and taken off of chromatin in a coordinated manner to ensure their proper function and ultimately to maintain faithful duplication and structural integrity of the genome. Biochemical studies have been able to elucidate many of the functions and functional interactions of major replication factors and other chromatin-bound proteins. These studies include co-immunoprecipitation analyses and ChIP analyses of human ORC and MCM proteins [32,33,35,36]. In order to determine changes in protein-chromatin interactions in direct relationship to DNA replication, these methodologies must rely on synchronization of a population of cells. In the present study, we were able to discern the distribution of replication-associated proteins, in the context of DNA replication, at the level of individual cells. While this is also possible with IF studies of interphase nuclei, the resolution of chromatin fibers is considerably higher; replication foci are about 1 Mb in size, which amounts to at least 10 replicons of approximately 100 kb in length in the space of 0.4 to 0.8 μm [17,18].

**Figure 7:**
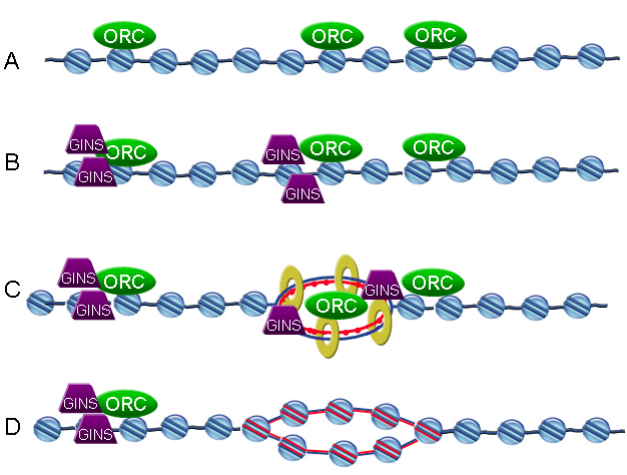
**Diagram summarizing the chromatin distribution of proteins analyzed in this study**. For simplicity, replication proteins that were not analyzed are not depicted in this diagram. Diagram is not drawn to scale. Light blue spheres represent nucleosomes and dark blue lines represent the DNA component of the chromatin fibers. We found that the relative distribution of ORC and GINS proteins changed in relation to DNA replication activity. (A) ORC proteins were found on chromatin before the binding of GINS and before DNA replication began. Some ORC complexes were found in close proximity on these fibers; they appeared as continuous streaks in digital photomicrographs. (B) GINS proteins were loaded onto chromatin after ORCs but before DNA replication began. (C) The number of ORC complexes per replication unit is not known, but it is presumed to be one; an ORC complex is depicted in the center of the replicon. The ORC complex that is shown associated with chromatin at the right edge of the replication track represents a dormant origin passively replicated later from a nearby active origin. The red dotted line represents newly synthesized DNA, yellow rings represent PCNA. (D) As replication proceeds, nucleosomes are assembled on the newly replicated DNA and ORC proteins are no longer visible on the fibers. GINS and PCNA also dissociate from sites were replication has been completed.

The increased resolution of chromatin fibers led us to some interesting, and previously unreported observations on the dynamics of ORC protein binding to chromatin. ORCs were found on chromatin fibers before replication began (Figure 7A); many chromatin fibers with no visible EdU incorporation contained densely bound ORCs. Once replication began, we found ORCs at sites of EdU incorporation as components of bead-like structures (Figure 7C). The apparent 'coating' of these structures with ORC proteins was not predicted from previous biochemical studies nor from IF studies of interphase nuclei. Based on ChIP studies, ORC proteins are only predicted to be found at replication origins. We also found that approximately 40% of EdU tracks (20-min labeling) were devoid of ORCs, suggesting that these proteins dissociated from the chromatin (Figure 7D). This trend toward dissociation of ORC proteins from replicated chromatin was even more pronounced with longer EdU incubation times (Figure 5). These results differ from those in the ChIP studies that reported that ORC1 dissociates from origins, but ORC2 does not [32,35]. At least one other study using IF showed a changing pattern of chromatin-associated ORC2 in nuclei during the cell cycle in several human cell lines [16], suggesting that ORC2 must dissociate from some chromatin sites.

There are at least two possible explanations for the different results found with ChIP and our methodology. It is possible that after DNA replication the association of ORCs with the chromatin is not as tight, making them more susceptible to extraction by the lysis buffer during the fiber extension process. Indeed, not all replication-associated proteins remain on the extended chromatin fibers; we have found that it is possible to visualize sites of Cdc6 binding on interphase nuclei, but Cdc6 was not found on the extended chromatin fibers (data not shown). In the case of ORC2, which unlike Cdc6 can be found on the extended chromatin fibers, there are reported changes in the type of nuclear associations observed for this protein during G_1 _and then again as cells enter S phase. For example, in human cells ORC2 is found almost exclusively in DNAse I-sensitive chromatin fractions during M and early G_1 _phases. Alternatively, ORC1 binds in the middle of G_1 _and has been reported to stay associated with DNAse I-resistant nuclear structures (structures that remain insoluble after DNAse treatment of chromatin) [37,38]. At the times that ORC1 was bound, levels of ORC2–5 complexes remained constant and a substantial amount (about half) was found associated with the DNAse I-resistant structures that ORC1 occupies. As S phase progressed, ORC1 dissociated and was degraded, while the levels of ORC2–5 decreased in the DNAse I-resistant and increased in DNAse I-sensitive fractions of the chromatin. The bead-like structures that are seen in this study at sites of active DNA replication may indeed be the nuclease resistance sites that have been noted in biochemical studies of ORC binding.

It is also possible that the ORCs that are dissociating from the replicated DNA are not from the origin where replication actually initiated, but are from licensed origins that never fired (so called 'dormant' origins). Two recent studies have shown the presence of dormant or 'backup' origins in human cells [39,40]. These dormant origins are sites that are licensed in G_1 _but are not initially activated during S phase. Instead, these dormant origins fire when cells are under replicative stress. Once these dormant origins are passively replicated from nearby origins, their associated pre-RCs are no longer necessary (and should not be activated within the same S phase) and are presumably removed from chromatin (Figures 7C and 7D).

In evaluating the extended chromatin fibers we also found that fluorescence signal from ORC1 and ORC2 antibodies did not always overlap. While it is true that ORC1 and ORC2 are both necessary components of a functional ORC complex they are not necessarily bound together on chromatin at all times. We base this assertion on several lines of evidence. First, as cells progress through S phase ORC1 is reportedly removed from chromatin and degraded in human cells, while ORC2 remains associated with centromeric regions [16]. As discussed above, ORC2 seems to be present on chromatin in G_1 _before ORC1. It should also be mentioned that ORC1 reportedly has non-replication associated functions; ORC1 has a role in transcriptional regulation of a subset of genes in both yeast [41] and human cells [42,43]. ORC1 can be co-immunoprecipitated in complexes that do not contain other ORC subunits [44]. Finally, experiments with antibodies to ORC2 either failed to co-immunoprecipitate [44] or only precipitated a fraction of chromatin bound ORC1 [35]. These co-immunoprecipitation results and our observations on extended chromatin fibers could indicate that there are substantial amounts of human ORC1 and ORC2 proteins that are bound to chromatin separately.

For GINS complex proteins, the data generated in our study largely reinforced information reported in biochemical studies. For example, GINS were loaded on chromatin after ORC proteins, but before DNA replication began, which is in agreement with the GINS complex role in initiation of DNA replication (Figure 7B). Also, GINS proteins were found overlapping sites of PCNA binding (Figure 7C). These findings are in agreement with published biochemical studies indicating that GINS are involved in elongation of DNA replication.

We were also able to study the dynamics of the association of GINS complex proteins as DNA replication progresses. It has been observed in human S phase cells that the GINS protein Psf2 co-immunoprecipitates with Cdc45 from both soluble and chromatin fractions [45]. It has also been reported that in *Xenopus *egg cell extracts the binding of GINS and Cdc45 to chromatin are mutually dependent [4]. Thus, we can infer the chromatin association of GINS from what is known about Cdc45. In human cells, Cdc45 is present in the nucleus during G_1 _but is not bound to chromatin until the G_1_/S border [45]. As cells proceed through S phase and into G_2_, Cdc45 can be seen distributed in a chromatin-associated punctate pattern. By metaphase, Cdc45 is found in a diffuse pattern throughout the nucleus. Based on these reported observations, we expect GINS to bind to pre-replication complexes only shortly before replication begins and to become dissociated from domains of replicated chromatin. Our results clearly show the latter; GINS proteins were no longer bound to EdU-labeled chromatin fibers (Figure 5). As for the former, the actual time span between the binding of GINS to chromatin and the start of DNA replication at a given site is not known. Our fibers do show many sites where GINS are bound, but no EdU signal is visible on the chromatin fiber. It is certainly possible that replication has initiated at these sites but the extent of EdU incorporation was not yet sufficient to generate a detectable signal. It is also possible that there is a measurable delay in the onset of replication after GINS are bound that can be detected with extended chromatin fiber technology, which affords simultaneous analysis of protein binding and replication activity at individual replicons. Using a higher concentration of EdU or an antibody-based amplification of the IF signal specific for the incorporation of DNA precursors at initiation sites could help discern which of these possibilities is correct.

In addition to analyzing the distribution of proteins, the methodology used in the present work allowed us to gain some new perspectives on the distribution of units of DNA replication. Aside from the measurement of distances between active sites of replication, we also found a new morphologically distinct structure. The bead-like structures that we observed on chromatin fibers with antibodies to replication-associated proteins and the labeling of EdU tracks could also be seen when we stained the DNA in the chromatin fibers with YOYO-1. As shown in Figure 4B, the bead-like structures are sites of more intense DNA staining that correspond with the position of PCNA and GINS, and almost appear to be sitting on top of the main DNA fiber. These are presumably sites where two double strands of newly replicated DNA are looped out from the main chromatin fiber and, as stated above, may be the nuclease-resistant sites that have been previously reported. In the nucleus these loops would have been supported by the three-dimensional lattice of nuclear matrix proteins that anchors the replication foci seen in interphase nuclei. These three-dimensional structures are altered as chromatin fibers are stretched and the supporting network is disrupted.

## Conclusion

The extended chromatin fibers analyzed in this study show the distribution and interrelationships of ORCs, PCNA and GINS complex proteins. GINS proteins are loaded on chromatin after ORC proteins, but before DNA replication begins. GINS proteins can also be found at sites of replication along with PCNA. These findings are in agreement with published biochemical studies indicating that GINS are involved in both the processes of initiation and elongation of DNA replication.

## Methods

### Preparation of extended chromatin fibers

The human cells used in these studies were NHF1-hTERT, a cell line derived from normal neonatal foreskin fibroblasts [46] and immortalized by ectopic expression of the catalytic subunit of telomerase [47]. NHF1-hTERT cells were incubated in 30 μM EdU (Invitrogen, Carlsbad CA, USA) for 20, 30, or 40 min and then washed in phosphate-buffered saline (PBS) and collected by trypsinization. Cells were pelleted and resuspended in warm hypotonic buffer (75 mM KCl) at 37°C for 20 min. Approximately 8,000 cells were cytospun for 4 min at 8,500 rpm (Cytofuge 2 from StatSpin) onto Superfrost Plus slides (Fisher Scientific) using a single-well gasket. After removal of excess fluid, 20 μl of a lysis buffer (25 mM Tris, pH 7.5, 0.5 M NaCl, 1% Triton X-100, and 0.2 M urea) [26] that contained 4',6-diamidino-2-phenylindole (DAPI; Sigma, 0.2 mg/ml) was added and the liquid was immediately covered with a 22 × 22 mm square coverslip. Lysis solution was allowed to evaporate overnight at room temperature protected from light. However, we found that it only took around 45 min for the lysis solution to recede from the edges of the slide (the areas that produced the best chromatin fibers). DAPI staining allowed for visualization of chromatin fibers and slides were chosen for further processing based on the quality of fibers.

### Immunofluorescence staining of chromatin fibers

For detection of proteins and sites of EdU incorporation, coverslips were carefully removed and slides were first incubated in KCM buffer (120 mM KCL, 20 mM NaCl, 10 mM Tris pH 7.5, 0.5 mM EDTA, 0.1% (v/v) Triton X-100 [48]) for 30 min. This solution was replaced with blocking/antibody dilution buffer (10% fetal bovine serum in KCM) for 30 min at room temperature. Blocking solution was removed and primary antibodies diluted with the antibody dilution buffer were added to slides for a 1-h incubation. Slides were then rinsed gently at a 45° angle with 1 ml of KCM buffer [48] followed by three five-min washes in KCM in coplin jars, with no agitation. Secondary antibodies were then added for 30 min, and washed as above. Extended fibers were fixed in 4% formalin/KCM, followed by a 5-min wash in PBS. Slides were then incubated for 30 min in EdU reaction solution (prepared as per the manufacturer's recommendation) followed by two 5-min washes in PBS. It should be noted that EdU was used for these studies because this molecule can be detected without denaturation of the DNA and possible disruption of chromatin-associated proteins. Some slides were stained with the DNA stain YOYO-1 iodide (1 μM in PBS) for 5 min in order to visualize all of the DNA fibers on the slide. These YOYO-1-stained slides could not be stained for EdU because the fluorescence spectra of the two fluorochromes were nearly identical. Slides were mounted using Prolong Gold (Invitrogen) and a 22 × 22 mm square coverslip.

Primary antibodies and dilutions used: ORC1 (N-17) diluted 1:100, PCNA (PC10) diluted 1:200, PCNA (FL-261) diluted 1:200, all from Santa Cruz Biotechnology Inc., Santa Cruz CA, USA; ORC2 diluted 1:200, Stressgen Bioreagents, Victoria BC, Canada; PSF1 (1:200) and PSF2 (1:2000), both from Abcam Inc., Cambridge MA, USA; H3 (1:1000) Novus Biologicals, Littleton CO, USA. Secondary antibodies were diluted 1:200 and were Alexa Fluor conjugates purchased from Invitrogen. All incubations were conducted in a moist chamber at room temperature. Microscopy was carried out using an Olympus FV500 confocal microscope using the sequential scanning mode.

We evaluated the distribution of two of the GINS proteins, Psf1 and Psf2. These proteins should map to the same location since they are part of the same complex. However, they are found on opposite ends of the GINS complex and therefore may not be equally accessible to antibody binding. For example, Psf1 reportedly binds to both chromatin and other replication proteins while Psf2 is primarily involved in stabilization of the GINS complex [19]. When both antibodies were incubated together, we found that Psf1 mapped to sites of Psf2 on extended chromatin fibers 85% of the time, indicating that they are indeed highlighting the same complex. They can also be seen overlapping sites of EdU incorporation in the same bead-like structures observed with ORC proteins (Additional file 1). The signal from Psf2 was consistently stronger than from Psf1 and displayed a wider surface of interaction with chromatin. This may be due to the above-mentioned differences in accessibility, or alternatively to the different affinities of the two antibodies. Since there was such a high degree of overlap however, for the remaining studies, we used the two proteins interchangeably, depending on the host species of the other antibodies that were used in co-incubations.

### Data analysis

Photomicrographic images were analyzed using Image J software [49] with UCSD [50] and McMaster Photobionics [51] plugins. Results were analyzed and graphed using Microsoft Excel 2002.

## Abbreviations

ChIP: chromatin immunoprecipitation; DAPI: 4',6-diamidino-2-phenylindole; EdU: 5-ethynyl-2'-deoxyuridine; FISH: fluorescence in situ hybridization; IF: immunofluorescence; MCM: minichromosome maintenance; ORC: origin recognition complex; PBS: phosphate-buffered saline; PCNA: proliferating cell nuclear antigen; pre-RC: pre-replicative complex; SEM: standard error of the mean.

## Competing interests

The authors declare that they have no competing interests.

## Authors' contributions

SMC performed all chromatin fiber experiments and data analyses, and also drafted the manuscript. PDC participated in the initial optimization in our laboratory of the extended chromatin fiber methodology. MC-S participated in data interpretation and manuscript editing. DGK was the initiator and director of this project. All authors participated in the design of this study and approved the final manuscript.

## Supplementary Material

Additional file 1 **Psf1 and Psf2 distribution on extended chromatin fibers**. Chromatin fibers were incubated with antibodies for the GINS complex proteins Psf1 (red signal) and Psf2 (blue signal) and for the DNA analog EdU (green signal). Two representative extended chromatin fibers are shown. The immunofluorescent signal for Psf2 was consistently stronger than that of Psf1 either due to the differences in epitope availabilities or antibody titers. Despite differences in the relative size of immunofluorescent signal, we found an 85% overlap of Psf2 with Psf1 (n = 8 fibers, 99 GINS sites) indicating that they were highlighting the same complex. Bars ≅ 25 μm (≅ 400 kb; bottom right of each panel). Click here for file
