# Supplementary material for: DNA replication and the GINS complex: localization on extended chromatin fibers
Source: Epigenetics Chromatin. 2009 May 14;2:6. doi: 10.1186/1756-8935-2-6 (PMC2686697; doi:10.1186/1756-8935-2-6)
Supplement: Additional file 1 — Psf1 and Psf2 distribution on extended chromatin fibers. Chromatin fibers were incubated with antibodies for the GINS complex proteins Psf1 (red signal) and Psf2 (blue signal) and for the DNA analog EdU (green signal). Two representative extended chromatin fibers are shown. The immunofluorescent signal for Psf2 was consistently stronger than that of Psf1 either due to the differences in epitope availabilities or antibody titers. Despite differences in the relative size of immunofluorescent signal, we found an 85% overlap of Psf2 with Psf1 (n = 8 fibers, 99 GINS sites) indicating that they were highlighting the same complex. Bars ≅ 25 μm (≅ 400 kb; bottom right of each panel). [file 1756-8935-2-6-S1.doc]

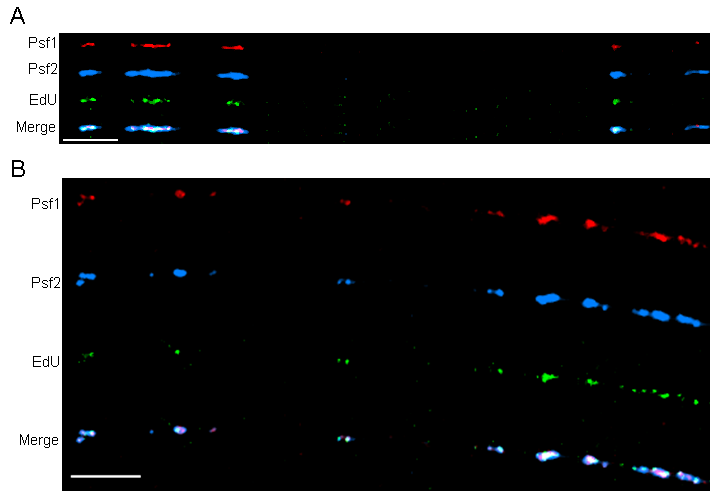


**Additional File 1.** **Psf1 and Psf2 distribution on extended chromatin fibers**. Chromatin fibers were incubated with antibodies for the GINS complex proteins Psf1 (red signal) and Psf2 (blue signal) and for the DNA analog EdU (green signal). Two representative extended chromatin fibers are shown. The immunofluorescent signal for Psf2 was consistently stronger than that of Psf1 either due to the differences in epitope availabilities or antibody titers. Despite differences in the relative size of immunofluorescent signal, we found an 85% overlap of Psf2 with Psf1 (n= 8 fibers, 99 GINS sites) indicating that they were highlighting the same complex. Bars @ 25 μm (≅400 kb; bottom right of each panel).
